# [Retracted] The role of Zeb1 in the pathogenesis of morbidly adherent placenta

**DOI:** 10.3892/mmr.2025.13493

**Published:** 2025-03-17

**Authors:** Na Li, Tian Yang, Wenqian Yu, Hao Liu, Chong Qiao, Caixia Liu

Mol Med Rep 20: 2812–2822, 2019; DOI: 10.3892/mmr.2019.10490

Following the publication of this paper, it was drawn to the Editor's attention by a concerned reader that certain of the flow cytometric assay data shown in Fig. 2C and 3C on p. 2817 and p. 2818 respectively were strikingly similar to data that had already been submitted for publication in a paper written by different authors at a different research institute.

Owing to the fact that the contentious data in the above article were already under consideration for publication prior to its submission to *Molecular Medicine Reports*, the Editor has decided that this paper should be retracted from the Journal. The authors were asked for an explanation to account for these concerns, but the Editorial Office did not receive a reply. The Editor apologizes to the readership for any inconvenience caused.

